# Quality of life and safety of a dietary intervention with French maritime pine bark extract (Pycnogenol^®^) in women with osteoporotic bone loss: a randomized, placebo-controlled double-blind study—Part II

**DOI:** 10.3389/fnut.2026.1924081

**Published:** 2026-10-12

**Authors:** Christian Scherg, Franca Genest, Jonas Maghary, Lothar Seefried, Petra Högger

**Affiliations:** 1 University of Würzburg, Institute of Pharmacy and Food Chemistry, Würzburg, Germany; 2 University of Würzburg, Osteology/Clinical Trial Unit, Würzburg, Germany

**Keywords:** bone metabolism, dietary intervention, osteoporosis, Pycnogenol^®^, quality of life, safety biomarkers

## Abstract

**Introduction:**

In the pre-therapeutic stage of osteopenia or clinically stable osteoporosis dietary supplements are frequently used, but often evidence for benefits and risks is sparse. The French maritime pine bark extract Pycnogenol^®^ has anti-inflammatory and antioxidative effects. Although beneficial impact on various health conditions has been recorded, no data regarding the impact on quality of life and safety of Pycnogenol^®^ supplementation in osteopenia and stable osteoporosis is available yet.

**Methods:**

In a randomized, double-blind, placebo-controlled clinical trial, 67 women received Pycnogenol^®^ (100 mg twice daily; *n* = 33) or placebo (*n* = 34) for 3 months, after which all participants took Pycnogenol^®^ for additional 3 months in an active-treatment extension phase. The quality of life was assessed by two validated questionnaires and biochemical safety indicators, including blood count, liver and kidney function, markers of inflammation and mineral metabolism were analyzed.

**Results:**

During the randomized placebo-controlled phase, the SF-36v2 bodily pain score improved in the Pycnogenol^®^ group while remaining unchanged under placebo. This effect persisted after adjustment for baseline values (*p* = 0.0106). A comparable difference was observed for vitality (*p* = 0.0282). The total QUALEFFO-41 score did not differ between the groups. In the QUALEFFO-41 pain domain, scores were lower after 6 months in the group treated continuously with Pycnogenol^®^ than in the group switched from placebo (*p* = 0.0231). During the intake of Pycnogenol^®^, the activities of the liver enzymes aspartate aminotransferase (AST) and alanine aminotransferase (ALT) remained stable, while a minor decrease in gamma glutamyl transferase (GGT) was significant (*p* = 0.0141) in the switched-over group. No significant changes were observed regarding blood count, kidney function (glomerular filtration rate GFR; blood urea) and inflammation (C-reactive protein; CRP). Serum phosphate concentrations declined under Pycnogenol^®^ within the reference range, reaching statistical significance after 6 months during the extension phase (*p* = 0.0039).

**Conclusion:**

Dietary supplementation with Pycnogenol^®^ maintained physiological blood count, markers of liver and kidney function, thus supporting the favorable safety of the plant extract. Additionally, participants receiving Pycnogenol^®^ experienced significant improvements in pain-related and vitality-related quality of life during the randomized placebo-controlled phase.

**Clinical trial registration:**

https://drks.de/search/de/trial/DRKS00026630, identifier DRKS00026630.

## 1 Introduction

Osteoporosis and osteopenia are prevalent metabolic bone disorders characterized by reduced bone mass and microarchitectural deterioration, leading to an increased risk of fractures. Osteoporosis, in particular, represents a major public health concern in aging populations (1). As bone mineral density (BMD) declines due to an imbalance between bone resorption and bone formation, affected individuals often suffer serious morbidity, reduced mobility, higher mortality, and decreased quality of life. Current therapeutic strategies for osteoporosis include antiresorptive agents and osteoanabolic therapies. In addition to pharmacological interventions, there is a growing interest in adjunct or alternative approaches using dietary supplements such as plant-derived extracts which may modulate bone turnover, reduce inflammation, and improve antioxidant status (2, 3).

Pycnogenol^®^ is a standardized extract from the bark of French maritime pine (*Pinus pinaster Ait.*) containing 65–75% oligomeric procyanidins and polyphenols such as catechin, ferulic acid, caffeic acid, and taxifolin. Preclinical studies have indicated its antioxidant and anti-inflammatory properties (4, 5). For example, in ovariectomized rat models of bone loss, Pycnogenol^®^ administration was associated with a balancing effect on excessive bone turnover, attenuation of bone loss, and improvements in bone strength (6–8). In human studies, Pycnogenol^®^ has also been shown to exert favorable effects on markers of cartilage degradation and inflammation in osteoarthritis (9, 10) and periodontitis (11).

Scientific data on clinical effects of Pycnogenol^®^ meanwhile amount to more than 12,000 participants in over 160 trials (10) and human safety data have been aggregated from multiple studies (12). The global frequency of adverse effects was low with gastrointestinal discomfort being the most reported event. As another safety aspect, routine biochemical laboratory parameters focusing on, e.g., liver and kidney function indicators as well as on hematological parameters have been investigated (13). While no pathologic alterations of any clinical parameters have been reported, data on long-term safety is rare because most studies span less than 6 months. Additionally, to the best of our knowledge, no bone-related parameters have been examined so far.

In a randomized, placebo-controlled double-blind clinical study the impact of a dietary intervention with Pycnogenol^®^ on quality of life and safety in women with low BMD was to be investigated. Quality of life in women with osteoporosis or osteopenia has been reported to be associated with bone microstructure and nutritional status (14). In the present evaluation, both a generic (SF-36v2) and a disease-specific questionnaire (QUALEFFO-41) were to be employed. The SF-36v2 questionnaire enables assessment of broad physical and mental health domains and allows comparison with populations without bone disease or normative data. In contrast, the QUALEFFO-41, developed for osteoporosis and especially for patients with vertebral fractures, focuses on domains directly affected by bone disease (15). Prior studies have shown that QUALEFFO-41 discriminates well between individuals with and without vertebral fractures, and correlates strongly with the SF-36 in various osteoporosis cohorts (16). By including both types of instruments, the study should detect not only disease-specific impairments but also how supplementation may affect global health, emotional well-being and social participation.

A thorough evaluation of routine laboratory and metabolic safety parameters over a six-month supplementation period with Pycnogenol^®^ sought to address markers of mineral metabolism such as serum calcium, phosphate, alkaline phosphatase (ALP), vitamin D, parathyroid hormone 1–84 (PTH) and intact fibroblast growth factor-23 (iFGF-23). The aim was to provide not only insights into bone turnover dynamics but also into homeostatic regulation of bone, kidneys, and endocrine feedback loops and all standard of care laboratory parameters essential for monitoring potential adverse effects of supplementation, particularly over a prolonged period.

We hypothesized that Pycnogenol^®^ supplementation could be beneficial for quality-of-life measures while being well tolerated, without adverse effects on laboratory indicators of mineral homeostasis, inflammation, liver or kidney function. The results were to be expected to provide for the first time insights into the risk–benefit profile of Pycnogenol^®^ in patients with osteopenia or clinically stable osteoporosis.

## 2 Materials and methods

### 2.1 Study design and participants

This monocenter clinical trial was conducted as a prospective, randomized, double-blind, placebo-controlled investigation with a two-arm parallel-group design comprising a three-month randomized, placebo-controlled phase followed by a three-month active-treatment extension phase. During the extension phase, participants initially allocated to placebo were switched to Pycnogenol^®^, so that all participants received the active treatment after 3 months. The study followed the principles of the Declaration of Helsinki for medical research involving human participants and was approved by the Ethics Committee of the University of Würzburg (approval no. 218/20). The trial was registered in the German Clinical Trials Register (DRKS00026630; September 14th, 2021). Between October 2021 and January 2024, a total of 67 participants were enrolled at the Osteology Department of Klinik König-Ludwig-Haus, University of Würzburg. All participants provided written informed consent before any study-related procedure was performed.

### 2.2 Intervention and monitoring

The investigational product was Pycnogenol^®^, a standardized French maritime pine bark extract meeting the quality requirements of the United States Pharmacopeia (USP) for dietary supplements outlined in the monograph “Pine extract.” Participants were allocated in a 1:1 ratio to receive either Pycnogenol^®^ capsules (100 mg twice daily) or identical appearing placebo capsules containing magnesium stearate, silicon dioxide, stearic acid, light brown dye and maltodextrin powder. Both the investigational product and the placebo were supplied by Horphag Research, Geneva, Switzerland. Group assignment followed a block randomization (blocks of ten) based on a computer-generated, rank-ordered list of random numbers created in Excel. Both participants and treating physicians (L.S., F.G., J.M.) remained blinded to the allocation, while the randomization code was held by the study evaluators (C.S. and P.H.), thereby maintaining the double-blind design. The trial comprised five visits: three on-site (V1, V3, V5) and two by telephone (V2, V4). At the baseline visit (V1), after informed consent and confirmation of eligibility, clinical and patient-related data were recorded, participants were randomized, and the intervention began. Participants also completed two quality-of-life questionnaires (QUALEFFO-41 and SF-36v2) and one nutrition questionnaire (17). At V3, 3 months later, clinical and laboratory parameters and questionnaires were reassessed; this visit also served as the prespecified interim analysis, which was based on the change in bone turnover markers of the first 40 participants reaching that milestone. The interim analysis was performed by the unblinded study evaluators (C.S. and P.H.), while participants and physicians involved in patient management remained blinded. The interim analysis was conducted solely to decide on continuation of the trial and involved no formal hypothesis testing, so that no adjustment of the significance level was required. Since the change in bone turnover markers was numerically more favorable under Pycnogenol^®^ than under placebo, the predefined criterion for continuation was met and the trial proceeded as planned, without modification of the sample size or the final analysis. After V3, participants initially assigned to placebo were switched to active treatment for the second three-month phase. The final on-site visit (V5), 6 months after initiation, repeated the clinical, laboratory, and questionnaire assessment. Between on-site visits, participants were contacted by telephone at V2 and V4 (6 weeks after V1 and V3, respectively) to record adverse events and confirm adherence.

The sample size was calculated to achieve a statistical power of 80% at a two-sided significance level of 5%, based on the bone turnover markers procollagen type I N propeptide (P1NP) and C-terminal telopeptide of type I collagen (CTX-1) as primary endpoints (results reported in a separate manuscript). Assuming a standard deviation of SD = ± 10 μg/L for P1NP and SD = ± 0.25 μg/L for CTX-1 (18), and clinically relevant changes of 20% corresponding to effect sizes of 0.7 and 0.8, the required sample sizes were 68 and 52 participants, respectively. Allowing for a drop-out rate of 10% and equal allocation to the treatment arms, a total of 76 participants was pursued. Quality of life was a prespecified secondary objective, and the analysis of standard laboratory markers was an explorative objective; the study was not powered to detect differences in these outcomes.

### 2.3 Inclusion and exclusion criteria

Eligible participants were men and women aged 18 years or older who met the criteria for osteopenia or osteoporosis according to the National German Guidelines for the Diagnosis and Treatment of Osteoporosis, defined by a ten-year fracture risk ≥ 20% (for postmenopausal women and men ≥ 60 years of age) together with a DXA T-score ≤ −1.0 at the total hip, femoral neck, or lumbar spine. Patients were not eligible if they were receiving antiosteoporotic pharmacotherapy – including any bisphosphonate, denosumab, strontium ranelate, teriparatide, or romosozumab – or had sustained an osteoporotic fracture within the 3 months preceding enrollment. Individuals with a current medical indication for osteoporosis treatment under the applicable national guidelines were likewise excluded, except where the patient declined such treatment, had a contraindication or an unfavorable risk–benefit ratio, or was pausing prior therapy to avoid side effects associated with long-term use. Concurrent intake of nutritional supplements was a further exclusion criterion.

### 2.4 Mineral metabolism markers and safety parameters

Serum samples were obtained to assess biochemical safety markers and indicators of mineral metabolism in line with applicable guidelines for osteoporosis management (19). Standardized laboratory testing, including parameters relevant to safety and efficacy of Pycnogenol^®^ supplementation, was conducted at the Orthopedic Clinic and collaborating laboratories. Laboratory data were obtained through routine measurements of the following parameters: alanine aminotransferase (ALT), aspartate aminotransferase (AST), gamma glutamyl transferase (GGT), C-reactive protein (CRP), leukocyte count, hemoglobin, urea, alkaline phosphatase (ALP), parathyroid hormone 1–84 (PTH), calcium, phosphate, intact fibroblast growth factor-23 (iFGF-23) and vitamin D. Renal function (glomerular filtration rate) was estimated from serum creatinine levels, age, and sex using the MDRD (Modification of Diet in Renal Disease) equation. Laboratory-specific reference intervals are given in the respective results tables and were used to assess whether individual values remained within the physiological range.

### 2.5 Quality-of-life questionnaires

Health-related quality of life (HRQoL) was assessed using the German language version of QUALEFFO-41 (Quality-of-Life Questionnaire of the International Osteoporosis Foundation / IOF) and the SF-36v2 (36-Item Short Form Health Survey version 2). The QUALEFFO-41 is a self-administered, disease-specific instrument developed for patients with osteoporotic vertebral fractures. The questionnaire comprises 41 items grouped into seven domains: pain, activities of daily living, jobs around the house, mobility, leisure and social activities, general health perception, and mental function. Domain and total scores are calculated by averaging item responses and linearly transforming to a 0–100 scale, where higher scores indicate worse quality of life (20, 21). Scoring followed the IOF scoring algorithm, version January 2020 (22). Missing values were disregarded; if more than 30% of the items of a domain or the whole questionnaire were missing, the respective domain score or the total score was excluded from the analysis. The SF-36v2 is a generic 36-item questionnaire covering eight domains: vitality, physical functioning, bodily pain, general health perception, physical role functioning, social role functioning, emotional role functioning, and mental health. Domain scores are aggregated into two summary indices: physical component summary (PCS) and mental component summary (MCS). These scores were compared against general adult population norms (mean = 50, SD = 10), based on a 2009 general US population representative sample (23). Each domain contains 2 to 10 questions, with a maximum total score of 100; higher scores indicate better HRQoL (24, 25). Missing item responses were replaced by the mean of the remaining items answered by the same respondent within the respective scale, provided that at least 50% of the items of that scale had been answered (26). Scales with more than 50% missing items were set to missing (26), and questionnaires with more than 25% missing items overall were excluded from the analysis.

### 2.6 Statistical analysis

All analyses were performed using the GraphPad^®^ Prism software, version 11 for windows (GraphPad Software, Inc., La Jolla, CA, United States). Prior to statistical comparisons, outliers were identified and excluded using the ROUT method implemented in GraphPad^®^ Prism, with a Q value of 1%. The outlier analysis was applied uniformly to all assessed parameters independently of treatment allocation. The procedure was not prespecified in the study protocol. Routine laboratory parameters and quality-of-life scores were analyzed using a mixed-effects model (REML) fitted with treatment group, time and their interaction as fixed effects and participant as a random effect, thereby including all available observations without requiring complete data at every visit. Pairwise comparisons within and between groups were adjusted for multiplicity using Tukey’s method within each parameter; no correction was applied across different parameters.

For parameters showing a between-group difference at month three for which an influence of baseline values could not be excluded, the randomized placebo-controlled phase was additionally analyzed by multiple linear regression, corresponding to an analysis of covariance (ANCOVA), with the three-month-value as dependent variable and the baseline value and treatment groups as independent variables. The regression coefficient for treatment group represents the baseline-adjusted treatment effect; 95% confidence intervals were determined by profile likelihood.

Spearman’s rank correlation coefficient was computed to evaluate relationships between variables, pooling observations from all time points and both treatment arms. Statistical significance was established at a two-sided alpha level of 0.05. As the quality of life was a prespecified secondary objective and the analysis of standard laboratory markers an explorative objective, these outcomes are reported without confirmatory claim.

## 3 Results

### 3.1 Participant flow and baseline characteristics

Following eligibility screening, 67 female patients were enrolled and randomized to receive either Pycnogenol^®^ (*n* = 33) or placebo (*n* = 34); the participant flow is illustrated in Figure 1 in accordance with the Consolidated Standards of Reporting Trials (CONSORT) guidelines. Dropout rates were low and balanced between groups (9.1% in the Pycnogenol^®^ vs. 8.8% in the placebo group). Baseline characteristics were comparable between the two arms: mean age (60.67 ± 13.12 years versus 63.09 ± 8.04 years) and BMI (24.78 ± 4.77 kg/m^2^ versus 24.19 ± 3.49 kg/m^2^) did not differ significantly, nor did any other variable, including smoking behavior, alcohol consumption, vegetarian diet, bone mineral density, fractures within the previous ten years (all sites and vertebral fractures), diagnosis of an inflammatory disease and analgesic use (Table 1). Two potentially treatment-related adverse events were recorded during the study. One participant in the Pycnogenol^®^ group reported dizziness and one participant in the placebo group experienced repeated vomiting; both events were rated as mild. Both participants discontinued the study. No moderate or serious adverse events or clinically relevant laboratory abnormalities were reported in either group.

**Figure 1:**
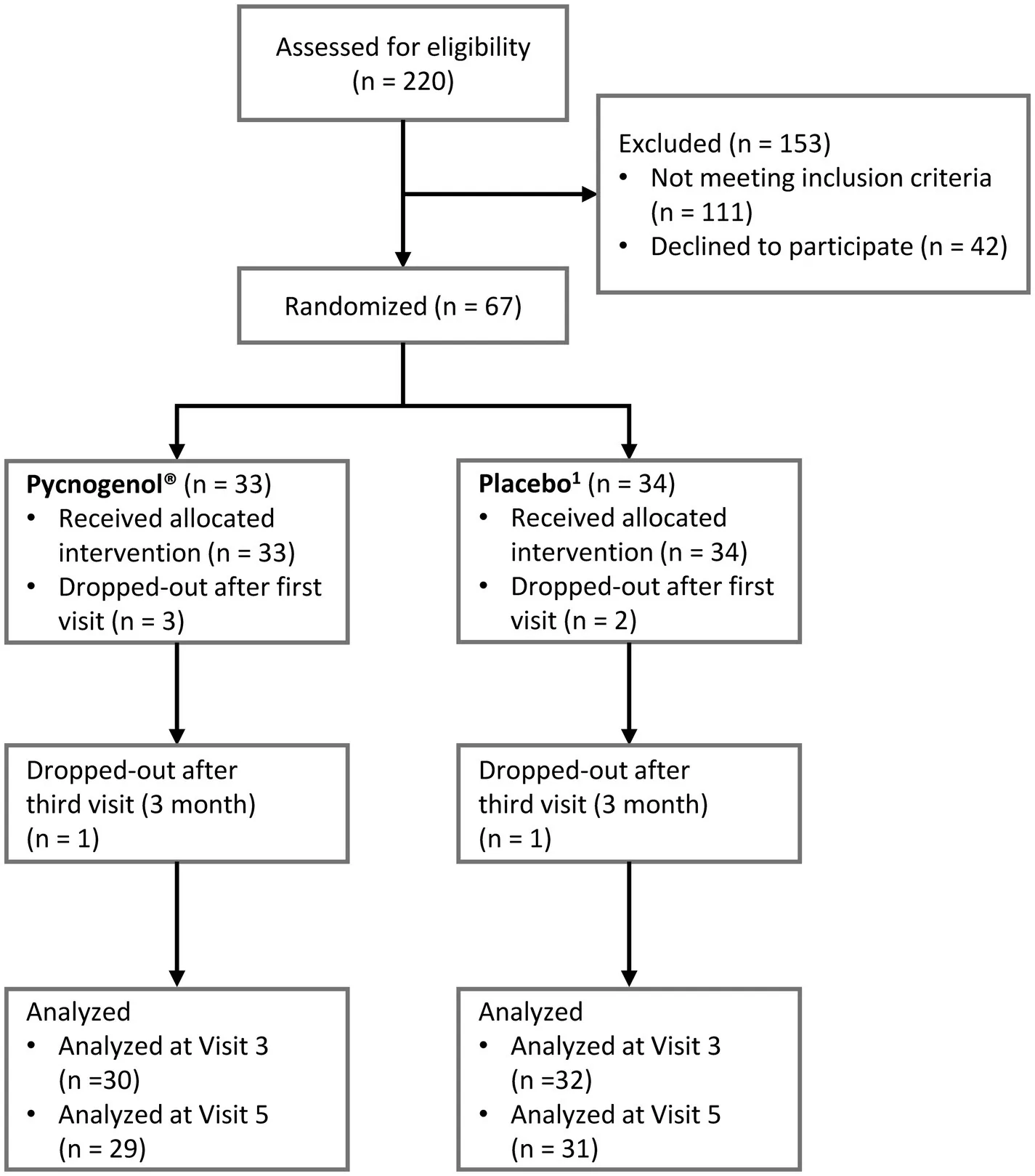
Consolidated Standards of Reporting Trials (CONSORT) flow chart illustrating the participant flow through the study, including enrollment, allocation to intervention groups (Pycnogenol^®^ and placebo), follow-up, and analysis. ^1^ 6-month value: switch to Pycnogenol^®^.

**Table 1:** Demographic and lifestyle characteristics of the study participants.

Variables	Pycnogenol^®^ group^1^ (*n* = 33)	Placebo group^1^ (*n* = 34)	*p*-value^2^
Age (years), mean ± SD	60.67 ± 13.12	63.09 ± 8.04	0.6115 (M)
BMI (kg/m^2^), mean ± SD	24.78 ± 4.77	24.19 ± 3.49	0.5967 (T)
L1-L4^3^ (T-Score), mean ± SD	−1.61 ± 0.95	−1.79 ± 1.12	0.5175 (T)
L femur (T-Score), mean ± SD	−1.41 ± 0.72	−1.70 ± 0.74	0.1326 (T)
Vegetarian diet, *n* (%)			>0.9999 (F)
Yes	3 (9.1)	2 (5.9)	
No	24 (72.7)	25 (73.5)	
Missing	6 (18.2)	7 (20.6)	
Smoking status, *n* (%)			0.7049 (F)
Yes	3 (9.1)	5 (14.7)	
No	24 (72.7)	23 (67.6)	
Missing	6 (18.2)	6 (17.6)	
Drinking status, *n* (%)			>0.9999 (F)
Yes	21 (63.6)	20 (58.8)	
No	6 (18.2)	7 (20.6)	
Missing	6 (18.2)	7 (20.6)	
Fractures ≤10 years ago, *n* (%)			0.2623 (F)
Yes	11 (33.3)	7 (20.6)	
No	18 (54.5)	24 (70.6)	
Missing	4 (12.1)	3 (8.8)	
Vertebral Fractures ≤ 10 years ago, *n* (%)			0.6059 (F)
Yes	2 (6.1)	1 (2.9)	
No	27 (81.8)	30 (88.2)	
Missing	4 (12.1)	3 (8.8)	
Inflammatory disease, *n* (%)			0.1772 (F)
Yes	7 (21.2)	13 (38.2)	
No	22 (66.7)	18 (52.9)	
Missing	4 (12.1)	3 (8.8)	
Analgesic use, *n* (%)			0.7501 (F)
Yes	5 (15.2)	7 (20.6)	
No	24 (72.7)	24 (70.6)	
Missing	4 (12.1)	3 (8.8)	

1 Differences in individual parameters are based on incomplete data sets.

2 Compared between groups.

Mann–Whitney test (M), two-tailed *t*-test (T), Fisher’s exact test (F). ^3^ L1-L4: lumbar vertebrae 1–4 (standard DXA region of interest for lumbar spine BMD) * Significant *p*-value for α < 0.05.

### 3.2 Mineral metabolism markers

Mineral metabolism markers are summarized in Table 2 and Figure 2. The number of observations excluded by the ROUT procedure for all laboratory parameters by treatment group and visit is detailed in Supplementary Table S1. Serum calcium concentrations remained constant in both study arms: 2.39 ± 0.09 mmol/L at baseline to 2.39 ± 0.11 mmol/L to 2.39 ± 0.13 mmol/L in the Pycnogenol^®^ group and 2.39 ± 0.08 mmol/L at baseline to 2.38 ± 0.09 mmol/L after 3 months of placebo and 2.39 ± 0.10 mmol/L after switching to Pycnogenol^®^. Stable average levels across the treatment period were also observed for ALP and vitamin D serum concentrations in both study arms (Table 2; Figure 2).

**Table 2:** Concentrations of mineral metabolism markers in serum, determined in the Pycnogenol^®^ and placebo group.

Parameter	Measurement time	Pycnogenol®		Placebo^2^		*p*-value ^b^	Mixed-effects model *p*-values ^c^
		Mean ± SD	*n* ^1^	Mean ± SD	*n* ^1^		
Calcium [mmol/L] Ref.: 2.1–2.6	Baseline (V1)	2.39 ± 0.09	30	2.39 ± 0.08	32	0.9589	Time: 0.9142 Group: 0.8526 Time × Group: 0.8514
	3 months (V3)	2.39 ± 0.11	30	2.38 ± 0.09	32	0.6497	
	*p*-value ^a^ vs. V1	0.9952		0.7810			
	6 months (V5)	2.39 ± 0.13	29	2.39 ± 0.10	31	0.9433	
	*p*-value ^a^ vs. V1	0.9953		0.9894			
	*p*-value ^a^ vs. V3	0.9979		0.8822			
Phosphate [mmol/L] Ref.: 0.6–1.8	Baseline (V1)	1.20 ± 0.14	30	1.15 ± 0.15	32	0.1237	Time: 0.0345 * Group: 0.6369 Time × Group: 0.0430 *
	3 months (V3)	1.15 ± 0.14	30	1.14 ± 0.14	32	0.7318	
	*p*-value ^a^ vs. V1	0.1160		0.9048			
	6 months (V5)	1.12 ± 0.15	29	1.15 ± 0.12	31	0.4286	
	*p*-value ^a^ vs. V1	0.0039 *		0.9807			
	*p*-value ^a^ vs. V3	0.4308		0.7308			
ALP [U/L] Ref.: 42–98	Baseline (V1)	77.61 ± 19.78	28	82.09 ± 20.24	32	0.3895	Time: 0.1649 Group: 0.8135 Time × Group: 0.7025
	3 months (V3)	76.61 ± 15.25	28	82.88 ± 17.14	32	0.1393	
	*p*-value ^a^ vs. V1	0.8881		0.9467			
	6 months (V5)	84.79 ± 25.37	29	84.03 ± 19.79	31	0.8979	
	*p*-value ^a^ vs. V1	0.7261		0.6700			
	*p*-value ^a^ vs. V3	0.2187		0.6224			
Vitamin D [ng/mL] Ref.: 20–40	Baseline (V1)	40.73 ± 14.36	30	40.09 ± 12.41	32	0.8523	Time: 0.5748 Group: 0.8547 Time × Group: 0.5627
	3 months (V3)	39.70 ± 15.10	30	40.63 ± 13.91	32	0.8032	
	*p*-value ^a^ vs. V1	0.5861		0.9439			
	6 months (V5)	38.93 ± 10.90	28	42.48 ± 10.39	31	0.2062	
	*p*-value ^a^ vs. V1	0.9997		0.5987			
	*p*-value ^a^ vs. V3	0.7736		0.6452			
PTH [pg/mL] Ref.: 14.9–56.9	Baseline (V1)	32.78 ± 9.37	29	35.15 ± 9.32	32	0.3275	Time: 0.0227 * Group: 0.1129 Time × Group: 0.3372
	3 months (V3)	31.48 ± 7.60	29	35.89 ± 9.23	31	0.0473*	
	*p*-value ^a^ vs. V1	0.8180		0.6463			
	6 months (V5)	33.77 ± 10.41	27	39.79 ± 13.07	27	0.0672	
	*p*-value ^a^ vs. V1	0.7904		0.0786			
	*p*-value ^a^ vs. V3	0.4304		0.1563			
Baseline-adjusted analysis, V1 → V3 ^d^; *β* (group) = −2.47; 95% CI = −5.39 to 0.45; *p* = 0.0959; *n* = 59							
iFGF-23 [pmol/L] Ref.: 0–5	Baseline (V1)	1.55 ± 1.20	29	1.36 ± 0.95	32	0.4996	Time: 0.4998 Group: 0.7579 Time × Group: 0.7093
	3 months (V3)	1.52 ± 0.98	27	1.59 ± 1.02	32	0.7625	
	*p*-value ^a^ vs. V1	0.8773		0.2878			
	6 months (V5)	1.57 ± 1.16	23	1.59 ± 1.13	30	0.9681	
	*p*-value ^a^ vs. V1	0.8930		0.5957			
	*p*-value ^a^ vs. V3	0.8937		0.9914			

1 Number of participants with a quantifiable value at the respective visit, after exclusion of outliers by the ROUT procedure (Q = 1%); *n* therefore varies between visits.

2 6-month value: Switch to Pycnogenol^®^.

Values are observed means ± SD. All *p-*values derive from one mixed-effects model (REML) per parameter, with treatment group, time and their interaction as fixed effects and participant as a random effect, using all available observations. Model estimates are therefore not derivable from the descriptive values shown. ^a^ Within-group comparisons versus V1 or V3; ^b^ between-group comparison at that visit, unadjusted for baseline. All pairwise comparisons were corrected for multiplicity using Tukey’s method. ^c^ Overall *p-*values for the fixed effects of the model. From V3 onward all participants received Pycnogenol^®^, so between-group comparisons at V5 reflect duration of exposure rather than treatment allocation and are descriptive. ^d^ Multiple linear regression for the randomized phase (V3 value as dependent variable, V1 value and treatment group as independent variables), corresponding to an analysis of covariance (ANCOVA). Performed for parameters showing a between-group difference at V3. *β* (group) is Pycnogenol^®^ minus placebo, with 95% CI by profile likelihood; participants with evaluable values at both V1 and V3. * Significant *p*-value for α < 0.05.

**Figure 2:**
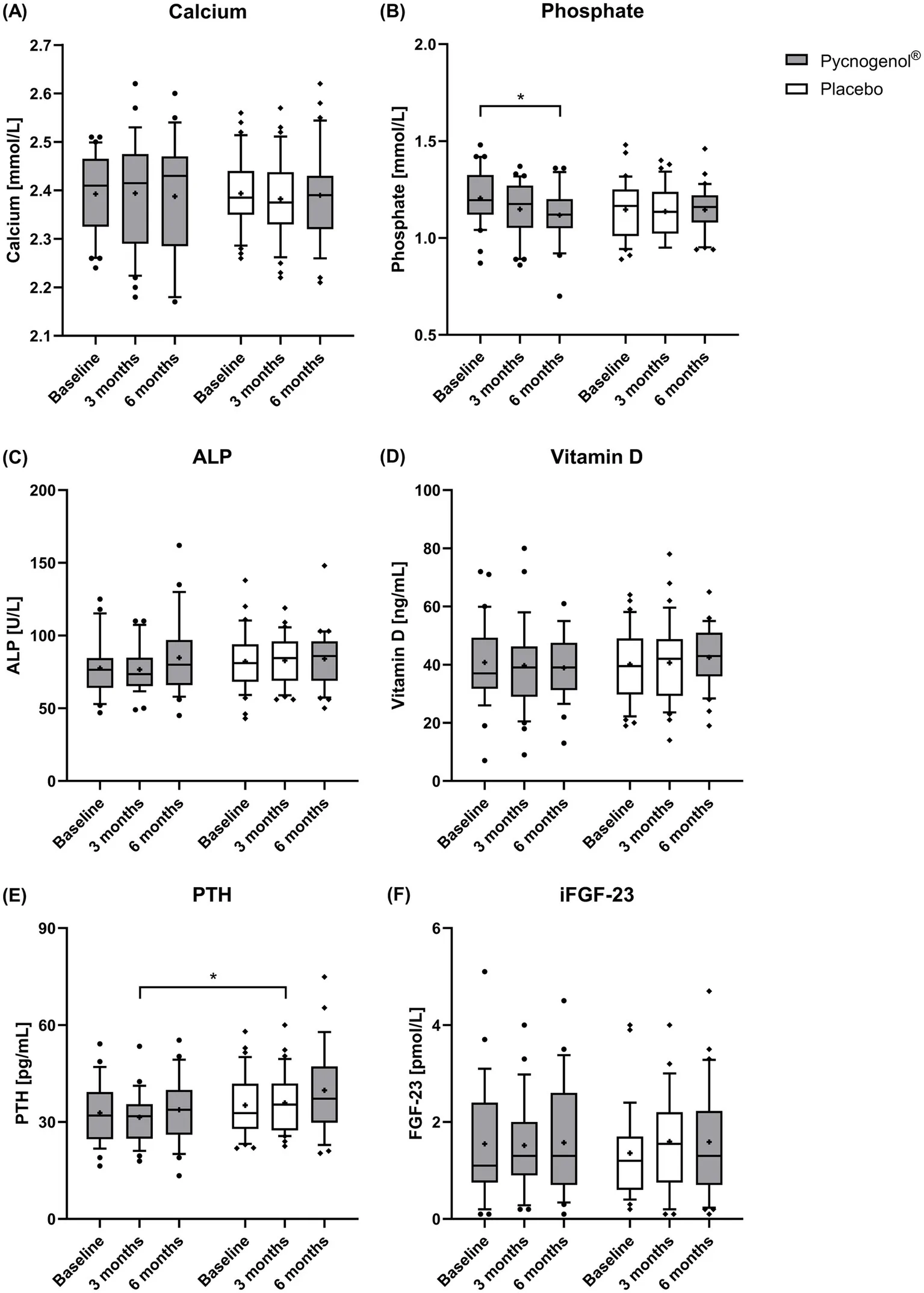
Mineral metabolism parameters (baseline, 3 and 6 months after start of intervention). Data are presented as Box-and-Whisker plot. Boxes show the interquartile range (Q1-Q3), with medians (line), means (+), whiskers (10th-90th percentiles), and outliers (circles/diamonds). Asterisks (*) indicate statistical significance at α < 0.05 for the comparison marked by the respective bracket. Numbers of participants per visit are listed in Table 2. **(A)** Calcium; **(B)** Phosphate, significant within-group difference in the Pycnogenol^®^ group between baseline and 6 months; **(C)** Alkaline phosphatase (ALP); **(D)** Vitamin D; **(E)** Parathyroid hormone 1–84 (PTH), significant between-group difference at 3 months; **(F)** Intact fibroblast growth factor-23 (iFGF-23).

For serum phosphate, the model indicated a significant treatment-by-time interaction (*p* = 0.0430), reflecting a decline in the Pycnogenol^®^ group that was absent under placebo. During the randomized placebo-controlled phase, concentrations decreased from 1.20 ± 0.14 mmol/L to 1.15 ± 0.14 mmol/L under Pycnogenol^®^ without reaching statistical significance (*p* = 0.1160). After 6 months of continuous Pycnogenol^®^ intake, the concentration was significantly lower compared to baseline (1.20 ± 0.14 mmol/L versus 1.12 ± 0.15 mmol/L, *p* = 0.0039). In the group switched from placebo, concentrations remained constant throughout, both during the first 3 months (1.15 ± 0.15 mmol/L to 1.14 ± 0.14 mmol/L) and after the switch to Pycnogenol^®^ (1.14 ± 0.14 mmol/L to 1.15 ± 0.12 mmol/L) (Table 2; Figure 2). Correlation analysis further identified a significant positive association between serum phosphate and the primary endpoint CTX-1 (*r* = 0.275, *p* < 0.001, *n* = 181).

At baseline, the serum PTH concentrations were not significantly different between the two groups. After 3 months, the concentration was significantly lower in the Pycnogenol^®^ group than in the placebo group (31.48 ± 7.60 pg/mL versus 35.89 ± 9.23 pg/mL, *p* = 0.0473); this difference was no longer detectable after adjustment for baseline values (*β* = −2.47; 95% CI = −5.39 to 0.45; *p* = 0.0959). Concentrations did not change within either group at any time point, and no between-group difference was seen after 6 months (Table 2; Figure 2).

The concentrations of iFGF-23 remained largely constant across both groups, with a tentative, non-significant increase (1.36 ± 0.95 pmol/L to 1.59 ± 1.02 pmol/L) observed after 3 months of placebo. Following the switch to Pycnogenol^®^, the concentrations stabilized at 1.59 ± 1.13 pmol/L (Table 2; Figure 2).

### 3.3 Safety parameters

All safety parameters remained within the respective reference ranges throughout the study in both groups (Table 3).

The activities of liver enzymes (AST and ALT) were largely constant in both study arms over the entire period, with no significant differences within or between the groups at any visit. For GGT, the model indicated a significant effect of time (*p* = 0.0017). Values were essentially unchanged during the randomized placebo-controlled phase in both arms, whereas lower concentrations were observed after 6 months. In the group switched from placebo to Pycnogenol^®^, GGT was significantly lower after 6 months compared to baseline (23.48 ± 7.90 U/L versus 20.29 ± 4.97 U/L, *p* = 0.0141) and after 3 months (24.72 ± 9.27 U/L versus 20.29 ± 4.97 U/L, *p* = 0.0472). A numerically comparable decline in the continuously treated Pycnogenol^®^ group (22.11 ± 7.28 U/L to 19.56 ± 4.75 U/L) did not reach statistical significance (*p* = 0.1550). No between-group differences were observed at any visit (Table 3).

Regarding values for renal function as an additional factor determining the safety of supplementation with the plant extract, both the GFR and the serum urea values remained virtually unchanged in both groups, with no significant differences within or between the groups at any timepoint (Table 3).

Analysis of blood count findings confirmed consistent hemoglobin serum concentrations and a steady leukocyte count in both study arms throughout the entire study period, with no significant differences within or between the groups (Table 3).

CRP concentrations differed between the groups from the outset. Baseline values were significantly higher in the Pycnogenol^®^ group (0.21 ± 0.20 mg/dL versus 0.09 ± 0.04 mg/dL, *p* = 0.0049), and this difference was also present after 3 months (0.25 ± 0.24 mg/dL versus 0.10 ± 0.00 mg/dL, *p* = 0.0041) and after 6 months (0.25 ± 0.25 mg/dL versus 0.13 ± 0.07 mg/dL, *p* = 0.0327), resulting in a significant overall group effect (*p* = 0.0004). No significant changes over time were observed within either group. In the baseline-adjusted analysis of the randomized placebo-controlled phase, no between-group difference was detectable (*β* = −0.011; 95% CI = −0.070 to 0.048; *p* = 0.7057).

**Table 3:** Concentrations of safety parameters in serum, determined in the Pycnogenol^®^ and placebo group.

Parameter	Measurement time	Pycnogenol^®^		Placebo^2^		*p*-value ^b^	Mixed-effects model *p*-values ^c^
		Mean ± SD	*n* ^1^	Mean ± SD	*n* ^1^		
AST [U/L] Ref.: 0–35	Baseline (V1)	22.52 ± 5.44	29	21.52 ± 5.10	31	0.4659	Time: 0.0535 Group: 0.9001 Time × Group: 0.5650
	3 months (V3)	21.66 ± 4.95	29	21.10 ± 4.71	31	0.6565	
	*p*-value ^a^ vs. V1	0.4152		0.7254			
	6 months (V5)	22.62 ± 5.07	29	22.65 ± 5.94	31	0.9863	
	*p*-value ^a^ vs. V1	0.9897		0.4322			
	*p*-value ^a^ vs. V3	0.2592		0.0830			
ALT [U/L] Ref.: 0–35	Baseline (V1)	21.43 ± 9.09	30	17.69 ± 5.02	29	0.0552	Time: 0.7370 Group: 0.5614 Time × Group: 0.0754
	3 months (V3)	20.07 ± 7.58	30	19.25 ± 6.81	32	0.6576	
	*p*-value ^a^ vs. V1	0.1941		0.9593			
	6 months (V5)	18.89 ± 6.91	28	20.39 ± 7.73	31	0.4363	
	*p*-value ^a^ vs. V1	0.4469		0.3161			
	*p*-value ^a^ vs. V3	0.9949		0.5697			
GGT [U/L] Ref.: 0–39	Baseline (V1)	22.11 ± 7.28	27	23.48 ± 7.90	27	0.5103	Time: 0.0017 * Group: 0.3042 Time × Group: 0.5674
	3 months (V3)	22.00 ± 8.27	26	24.72 ± 9.27	29	0.2545	
	*p*-value ^a^ vs. V1	0.9469		0.9942			
	6 months (V5)	19.56 ± 4.75	25	20.29 ± 4.97	24	0.6010	
	*p*-value ^a^ vs. V1	0.1550		0.0141 *			
	*p*-value ^a^ vs. V3	0.3591		0.0472 *			
GFR [mL/min] Ref.: 80–140	Baseline (V1)	86.83 ± 18.32	30	80.77 ± 9.44	30	0.1141	Time: 0.2352 Group: 0.3188 Time × Group: 0.6223
	3 months (V3)	86.77 ± 16.37	30	80.65 ± 11.33	31	0.0965	
	*p*-value ^a^ vs. V1	0.9993		0.7850			
	6 months (V5)	83.21 ± 18.25	29	81.07 ± 15.92	30	0.6336	
	*p*-value ^a^ vs. V1	0.4829		0.4512			
	*p*-value ^a^ vs. V3	0.4196		0.7992			
Urea [mmol/L] Ref.: 1.7–7.5	Baseline (V1)	5.76 ± 1.34	30	5.34 ± 1.25	32	0.2053	Time: 0.6409 Group: 0.4792 Time × Group: 0.3765
	3 months (V3)	5.61 ± 1.08	30	5.49 ± 1.29	32	0.6859	
	*p*-value ^a^ vs. V1	0.7654		0.7697			
	6 months (V5)	5.49 ± 1.17	29	5.43 ± 1.21	31	0.8355	
	*p*-value ^a^ vs. V1	0.2290		0.9306			
	*p*-value ^a^ vs. V3	0.7215		0.9106			
Hemoglobin [g/dL] Ref.: 12.0–16.0	Baseline (V1)	13.57 ± 1.02	30	13.81 ± 0.76	32	0.2958	Time: 0.1961 Group: 0.3602 Time × Group: 0.6991
	3 months (V3)	13.46 ± 1.08	30	13.68 ± 0.80	32	0.3595	
	*p*-value ^a^ vs. V1	0.5296		0.4884			
	6 months (V5)	13.51 ± 0.94	29	13.64 ± 0.82	31	0.5772	
	*p*-value ^a^ vs. V1	0.6674		0.2311			
	*p*-value ^a^ vs. V3	0.8365		0.8722			
Leukocytes [103/μL] Ref.: 4.0–10.0	Baseline (V1)	6.11 ± 1.58	28	6.44 ± 1.95	32	0.4710	Time: 0.7221 Group: 0.6508 Time × Group: 0.7756
	3 months (V3)	5.97 ± 1.44	28	6.29 ± 1.89	32	0.4579	
	*p*-value ^a^ vs. V1	0.8571		0.8391			
	6 months (V5)	6.25 ± 1.48	27	6.31 ± 1.37	31	0.8706	
	*p*-value ^a^ vs. V1	0.9404		0.7945			
	*p*-value ^a^ vs. V3	0.5206		0.9822			
CRP [mg/dL] Ref.: 0.0–0.8	Baseline (V1)	0.21 ± 0.20	27	0.09 ± 0.04	24	0.0049 *	Time: 0.3186 Group: 0.0004 * Time × Group: 0.8039
	3 months (V3)	0.25 ± 0.24	26	0.10 ± 0.00	23	0.0041 *	
	*p*-value ^a^ vs. V1	0.9819		0.1865			
	6 months (V5)	0.25 ± 0.25	25	0.13 ± 0.07	27	0.0327 *	
	*p*-value ^a^ vs. V1	0.7623		0.1024			
	*p*-value ^a^ vs. V3	>0.9999		0.2865			
Baseline-adjusted analysis, V1 → V3 ^d^; *β* (group) = −0.011; 95% CI = −0.070 to 0.048; *p* = 0.7057; *n* = 44							

1 Number of participants with a quantifiable value at the respective visit, after exclusion of outliers by the ROUT procedure (Q = 1%); *n* therefore varies between visits.

2 6-month value: Switch to Pycnogenol^®^.

Values are observed means ± SD. All *p-*values derive from one mixed-effects model (REML) per parameter, with treatment group, time and their interaction as fixed effects and participant as a random effect, using all available observations. Model estimates are therefore not derivable from the descriptive values shown. ^a^ Within-group comparisons versus V1 or V3; ^b^ between-group comparison at that visit, unadjusted for baseline. All pairwise comparisons were corrected for multiplicity using Tukey’s method. ^c^ Overall *p-*values for the fixed effects of the model. From V3 onward all participants received Pycnogenol^®^, so between-group comparisons at V5 reflect duration of exposure rather than treatment allocation and are descriptive. ^d^ Multiple linear regression for the randomized phase (V3 value as dependent variable, V1 value and treatment group as independent variables), corresponding to an analysis of covariance (ANCOVA). Performed for parameters showing a between-group difference at V3. *β* (group) is Pycnogenol^®^ minus placebo, with 95% CI by profile likelihood; participants with evaluable values at both V1 and V3. * Significant *p*-value for α < 0.05.

### 3.4 Quality-of-life questionnaires

The total QUALEFFO-41 score, the pain domains of both questionnaires and the SF-36v2 vitality domain are summarized in Table 4; all remaining domains are detailed in Supplementary Tables S4, S5. The completeness of the questionnaires is reported for each domain by treatment group and visit in Supplementary Tables S2, S3.

At baseline, the total QUALEFFO-41 score, in which a lower score signifies a higher quality of life, was not significantly different between the two groups, though numerically lower in the Pycnogenol^®^ group. During the randomized placebo-controlled phase, no significant between-group difference was observed after 3 months (19.20 ± 11.06 versus 26.34 ± 15.37, *p* = 0.0592), and the baseline-adjusted analysis likewise showed no treatment effect (*β* = −1.54; 95% CI = −4.96 to 1.88; *p* = 0.3692). Scores remained essentially unchanged within both groups throughout the study (Table 4; Figure 3).

**Table 4:** QUALEFFO-41 and SF-36v2 Scores recorded by the study participants.

Domain	Measurement time	Pycnogenol^®^		Placebo^2^		*p*-value ^b^	Mixed-effects model *p*-values ^c^
		Mean ± SD	*n* ^1^	Mean ± SD	*n* ^1^		
QUALEFFO-41 Total	Baseline (V1)	22.26 ± 13.76	29	28.83 ± 15.98	32	0.0895	Time: 0.2194 Group: 0.0858 Time × Group: 0.7803
	3 months (V3)	19.20 ± 11.06	25	26.34 ± 15.37	27	0.0592	
	*p*-value ^a^ vs. V1	0.4127		0.7006			
	6 months (V5)	22.09 ± 13.91	21	26.63 ± 15.96	29	0.2895	
	*p*-value ^a^ vs. V1	0.5768		0.5336			
	*p*-value ^a^ vs. V3	0.6025		0.9217			
Baseline-adjusted analysis, V1 → V3 ^d^; *β* (group) = −1.54; 95% CI = −4.96 to 1.88; *p* = 0.3692; *n* = 52							
QUALEFFO-41 Pain	Baseline (V1)	27.54 ± 24.99	29	38.30 ± 24.85	32	0.0976	Time: 0.5106 Group: 0.0242 * Time × Group: 0.5285
	3 months (V3)	25.00 ± 25.65	26	36.67 ± 26.64	27	0.1105	
	*p*-value ^a^ vs. V1	0.7179		0.9604			
	6 months (V5)	21.85 ± 20.81	21	37.50 ± 26.29	29	0.0231 *	
	*p*-value ^a^ vs. V1	0.0779		0.9947			
	*p*-value ^a^ vs. V3	0.9860		0.9981			
Baseline-adjusted analysis, V1 → V3 ^d^; *β* (group) = −4.52; 95% CI = −15.07 to 6.03; *p* = 0.3935; *n* = 53							
SF-36 Vitality	Baseline (V1)	56.07 ± 21.96	28	53.71 ± 19.15	31	0.6630	Time: 0.5134 Group: 0.2488 Time × Group: 0.1340
	3 months (V3)	64.62 ± 19.07	26	54.36 ± 18.09	28	0.0482 *	
	*p*-value ^a^ vs. V1	0.1017		0.6645			
	6 months (V5)	60.91 ± 20.91	22	54.46 ± 20.65	28	0.2825	
	*p*-value ^a^ vs. V1	0.9493		>0.9999			
	*p*-value ^a^ vs. V3	0.3557		0.7443			
Baseline-adjusted analysis, V1 → V3 ^d^; *β* (group) = 8.47; 95% CI = 0.94 to 15.99; *p* = 0.0282 *; *n* = 52							
SF-36 Bodily Pain	Baseline (V1)	57.46 ± 23.64	28	55.35 ± 25.05	31	0.7406	Time: 0.4378 Group: 0.0535 Time × Group: 0.0102 *
	3 months (V3)	68.07 ± 27.16	28	51.93 ± 26.47	28	0.0284 *	
	*p*-value ^a^ vs. V1	0.0287 *		0.6157			
	6 months (V5)	69.41 ± 24.90	22	51.75 ± 21.06	28	0.0110 *	
	*p*-value ^a^ vs. V1	0.0921		0.3386			
	*p*-value ^a^ vs. V3	0.7375		0.8603			
Baseline-adjusted analysis, V1 → V3 ^d^; *β* (group) = 13.77; 95% CI = 3.36 to 24.19; *p* = 0.0106 *; *n* = 53							

1 Number of participants with an evaluable domain score at the respective visit, after exclusion of incomplete questionnaires according to the scoring rules described in the Methods section and of outliers by the ROUT procedure (Q = 1%); *n* therefore varies between visits.

2 6-month value: Switch to Pycnogenol^®^.

In the QUALEFFO-41 questionnaire, lower scores signify a higher quality of life, in the SF-36v2 questionnaire, higher scores indicate a higher quality of life. Values are observed means ± SD. All *p-*values derive from one mixed-effects model (REML) per domain, with treatment group, time and their interaction as fixed effects and participant as a random effect, using all available observations. Model estimates are therefore not derivable from the descriptive values shown. ^a^ Within-group comparisons versus V1 or V3; ^b^ between-group comparison at that visit, unadjusted for baseline. All pairwise comparisons were corrected for multiplicity using Tukey’s method. ^c^ Overall *p-*values for the fixed effects of the model. From V3 onward all participants received Pycnogenol^®^, so between-group comparisons at V5 reflect duration of exposure rather than treatment allocation and are descriptive. ^d^ Multiple linear regression for the randomized phase (V3 score as dependent variable, V1 score and treatment group as independent variables), corresponding to an analysis of covariance (ANCOVA). *β* (group) is Pycnogenol^®^ minus placebo, with 95% CI by profile likelihood; participants with evaluable scores at both V1 and V3. * Significant *p*-value for α < 0.05.

**Figure 3:**
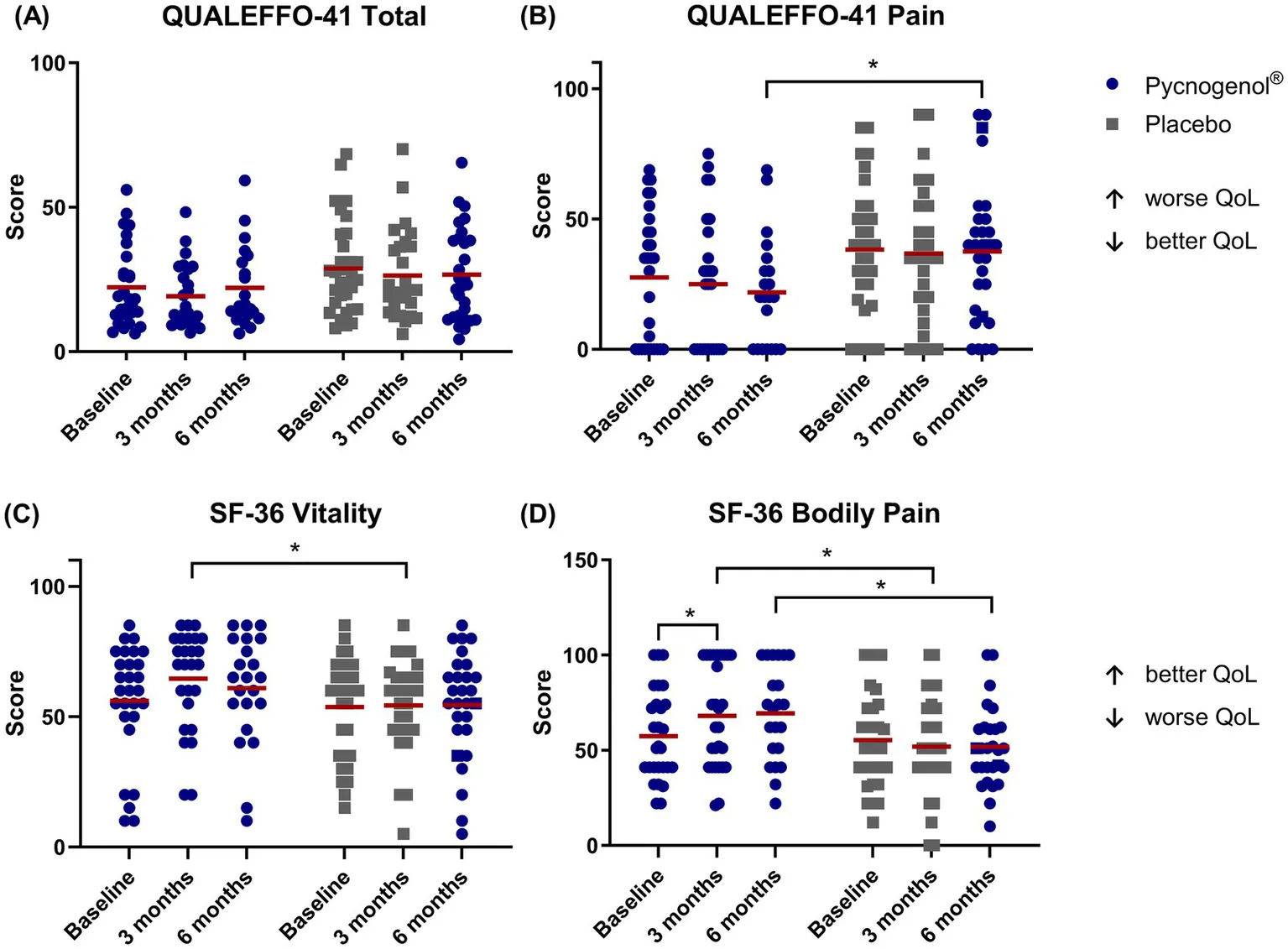
Quality of life questionnaires (baseline, 3 and 6 months after start of intervention). Data are presented as Scatter dot plot. Blue circles denote the Pycnogenol^®^ group; grey squares represent the placebo group. Mean values are indicated by a red horizontal line. Asterisks (*) indicate statistical significance at α < 0.05 for the comparison marked by the respective bracket. Numbers of participants per visit are listed in Table 4. **(A)** QUALEFFO-41 Total Score; **(B)** QUALEFFO-41 Pain, significant between-group difference at 6 months; **(C)** SF-36 Vitality, significant between-group difference at 3 months; **(D)** SF-36 Bodily Pain, significant within-group difference in the Pycnogenol^®^ group between baseline and 3 months and significant between-group differences at 3 and 6 months.

In the pain domain of the QUALEFFO-41, scores were also numerically lower in the Pycnogenol^®^ group at baseline. No significant between-group difference was detected during the randomized phase, either in the unadjusted comparison after 3 months (25.00 ± 25.65 versus 36.67 ± 26.64, *p* = 0.1105) or after adjustment for baseline values (*β* = −4.52; 95% CI = −15.07 to 6.03; *p* = 0.3935). At the end of the 3-month extension phase, scores were significantly lower in the group that had received Pycnogenol^®^ over 6 months compared to the group switched from placebo after 3 months (21.85 ± 20.81 versus 37.50 ± 26.29, *p* = 0.0231), and the mixed-effects model indicated an overall group effect (*p* = 0.0242). As all participants received Pycnogenol^®^ from month three onwards, this comparison reflected the duration of exposure. The within-group decrease in the group continuously taking Pycnogenol^®^ did not reach statistical significance (27.54 ± 24.99 to 21.85 ± 20.81, *p* = 0.0779) (Table 4; Figure 3).

Due to missing data, the Physical Component Summary (PCS) and Mental Component Summary (MCS) of the SF-36v2 questionnaire could not be reliably determined. The pain domains of both instruments were inversely correlated (*r* = −0.660, *p* < 0.0001, *n* = 158), and both the SF-36v2 bodily pain and vitality domains correlated with the QUALEFFO-41 total score (*r* = −0.751, *p* < 0.0001, *n* = 158 and r = −0.715, *p* < 0.0001, *n* = 157, respectively).

In the bodily pain domain of the SF-36v2, scores increased significantly in the Pycnogenol^®^ group during the randomized placebo-controlled phase (57.46 ± 23.64 versus 68.07 ± 27.16, *p* = 0.0287), while remaining unchanged under placebo (55.35 ± 25.05 versus 51.93 ± 26.47, *p* = 0.6157). After 3 months, scores were significantly higher in the Pycnogenol^®^ group than in the placebo group (68.07 ± 27.16 versus 51.93 ± 26.47, *p* = 0.0284), and this difference persisted after adjustment for baseline values (*β* = 13.77; 95% CI = 3.36 to 24.19; *p* = 0.0106). The mixed-effects model further indicated a significant treatment-by-time interaction (*p* = 0.0102). During the extension phase, scores remained stable in both arms, and the difference between the group continuously taking Pycnogenol^®^ and the switched-over group was still present after 6 months (69.41 ± 24.90 versus 51.75 ± 21.06, *p* = 0.0110) (Table 4; Figure 3).

In the vitality domain, scores were significantly higher in the Pycnogenol^®^ group than in the placebo group after 3 months (64.62 ± 19.07 versus 54.36 ± 18.09, *p* = 0.0482), and this difference was confirmed in the baseline-adjusted analysis (*β* = 8.47; 95% CI = 0.94 to 15.99; *p* = 0.0282). The within-group change in the Pycnogenol^®^ group from baseline to 3 months did not reach statistical significance (56.07 ± 21.96 versus 64.62 ± 19.07, *p* = 0.1017), and no significant difference was observed after 6 months (Table 4; Figure 3).

In the remaining domains, no changes over time were observed in either group. In the QUALEFFO-41 domain jobs around the house, scores were significantly lower in the Pycnogenol^®^ group after 3 and 6 months, but this difference was not confirmed after adjustment for baseline values (*β* = −4.21; 95% CI = −8.76 to 0.35; *p* = 0.0692) and was therefore attributable to lower baseline scores in this group. In the QUALEFFO-41 domain mental function, baseline scores already differed significantly in favor of the Pycnogenol^®^ group, with no significant differences at any later visit (Supplementary Table S4). In the SF-36v2 domain emotional role functioning, responses showed a pronounced ceiling effect; after outlier removal, all remaining values in the Pycnogenol^®^ group were at maximum score, leaving no variance in this group, so that between-group comparisons in this domain are not interpretable (Supplementary Table S5).

## 4 Discussion

The present study was the first to investigate a quality-of-life evaluation and extended safety parameters of a six-month dietary supplementation with Pycnogenol^®^, a procyanidin-rich French maritime pine bark extract, in a cohort of 67 women with osteopenia or stable osteoporosis. The trial comprised a three-month randomized, double-blind, placebo-controlled phase followed by a three- month active-treatment extension.

To the best of our knowledge, extended safety parameters in women with low BMD have not been reported after intake of Pycnogenol^®^, although certain aspects of other routine markers have been reported for, e.g., patients with stable heart disease (13) and chronic venous insufficiency (27). Our findings demonstrated that Pycnogenol^®^ supplementation had no significant impact on serum calcium or alkaline phosphatase levels, which were all within the reference ranges. This is largely consistent with earlier animal studies, where the French pine bark extract did not alter serum mineral concentrations or ALP activity (6). A decrease in serum phosphate concentrations was observed under Pycnogenol^®^, and the two study arms differed significantly in their course over the study period. The extent of change was limited, with mean serum phosphate concentrations declining from 1.20 ± 0.14 mmol/L to 1.12 ± 0.15 mmol/L after 6 months, whereas the difference during the randomized placebo-controlled phase did not reach statistical significance. Notably, the significant positive correlation between serum phosphate and the bone resorption marker CTX-1 observed in the present study (*r* = 0.275, *p* < 0.001, *n* = 181) suggests a functional link between phosphate metabolism and bone degradation. Although higher phosphate levels above a threshold of 1.20 mmol/L have been suggested to be associated with elevated fracture risks in women in the Rotterdam study (28), there is yet insufficient evidence to assume that a reduction of phosphate levels would contribute to reducing fracture risk. Serum PTH concentrations were significantly lower after 3 months of Pycnogenol^®^ intake than after placebo, while remaining unchanged within both groups and well within the reference range. This difference was no longer detectable after adjustment for baseline values and should therefore not be interpreted as an established treatment effect. The direction of the observation is nevertheless of interest, since PTH is a principal stimulus of osteoclastic bone resorption (29, 30), and lower concentrations would be consistent with a reduced bone resorption activity. Given the absence of calcium fluctuations and the lack of a consistent change in vitamin D—the two principal regulators of PTH secretion—the observed difference is unlikely to reflect classical homeostatic regulation (31, 32). Instead, it may be explained by other factors, including circadian fluctuations (33, 34), patients’ feeding status (35), and biological intra-individual variability (36).

Health-related quality of life (HRQoL) is significantly reduced in patients with osteoporosis compared to individuals with normal bone mineral density (37, 38). In women with osteopenia or osteoporosis, HRQoL correlated with the bone density and the nutritional status (14). Therefore, measuring quality of life as a secondary endpoint in clinical trials of interventions for osteoporosis is warranted (21). The disease-specific QUALEFFO-41 questionnaire has been shown to be a valid and reliable instrument for assessing health-related quality of life in patients with osteoporosis (15, 39, 40). Moreover, QUALEFFO-41 domains correlate significantly with corresponding domains of the SF-36v2 questionnaire, supporting its concurrent validity with a generic health status instrument (16). Our data corroborated this relationship, with strong inverse correlations observed between corresponding domains as well as between the SF-36v2 pain and vitality domains and the QUALEFFO-41 total score, consistent with the opposite scoring conventions of the two instruments.

The present study showed significant improvements in two domains of the SF-36v2 during the randomized placebo-controlled phase. In the bodily pain domain, scores increased under Pycnogenol^®^ while remaining unchanged under placebo and the difference persisted after adjustment for baseline values. The two arms also differed significantly in their course over the study period. A comparable, though smaller difference was observed for vitality. These findings are relevant since pain distinctly impairs quality of life. Pain does not only arise as a consequence of fragility fractures, but also from activation of nociceptive nerve fibers during osteoclastic hyperactivity, changes in bone innervation and muscular and skeletal changes. This contributes to functional limitations and promotes disability (41). Notably, after just 3 months of supplementation with Pycnogenol^®^, the SF-36v2 revealed a significant superiority of the supplement over the placebo group in the pain and vitality subscales. In contrast, a supplementation with green tea polyphenols of 500 mg/day for 24 weeks did not affect quality of life in postmenopausal osteopenic women, as assessed by the SF-36v2 questionnaire (42). Furthermore, a standardized hop extract combined with calcium and vitamin D3 supplementation, resulted in a significant increase in the SF-36v2 physical functioning score compared to placebo, but showed no significant effect in the pain domain (43). These findings suggest that while certain plant-based interventions may improve specific aspects of HRQoL, they do not uniformly impact all domains. The positive impact of Pycnogenol^®^ on pain scores is likely related to its established anti-inflammatory and antioxidant activities (10).

The disease-specific QUALEFFO-41 did not reproduce these findings during the randomized phase. Neither the total score nor the pain domain differed between the groups after 3 months once baseline values were taken into account. The pain domain showed a decrease under Pycnogenol^®^ over 6 months that did not reach statistical significance (*p* = 0.0779), and scores were lower than in the group switched from placebo at the same time point. As all participants received Pycnogenol^®^ from month three onwards, this latter comparison reflected the duration of exposure rather than treatment allocation. A recurring feature of the present dataset was that the Pycnogenol^®^ group started with numerically more favorable scores in several domains. The baseline-adjusted analyses showed that the apparent group differences in these domains were largely attributable to this imbalance. In contrast, the improvement in the SF-36v2 bodily pain and vitality scores persisted after baseline adjustment.

Data completeness differed between the two arms, with a higher proportion of unevaluable questionnaires in the Pycnogenol^®^ group at later visits (Supplementary Tables S2, S3). Selective drop-out of participants experiencing less benefit cannot be excluded on this basis. The mixed-effects model used all available observations and yields unbiased estimates as long as data is missing at random. The baseline-adjusted analyses, based on a different subset of participants, led to the same conclusions. Nevertheless, the possibility of selection bias remains and should be considered when interpreting the questionnaire results.

For the same reason, physical and mental component summary scores of the SF-36v2 questionnaire were not calculated. The imputation rule applied here operates at the item level within a scale and does not extend to entire scales. Summary scores require valid values for all eight subscales (26). Several participants lacked at least one complete scale, so that any summary score analysis would have been restricted to a substantially smaller subgroup. Work on the analogously constructed SF-12 summary scores illustrates how rapidly imputed estimates deteriorate as missing components accumulate, with agreement between imputed and true scores falling below an intraclass correlation of 0.50 once four or more key items are absent (26). Since a missing scale corresponds to the loss of several items at once, the analysis was restricted to the eight scale scores.

Safety parameters reflecting liver and kidney function were within the reference ranges and generally did not change under supplementation of 200 mg/day Pycnogenol®. AST and ALT activities remained essentially stable throughout the study in both treatment arms, a finding which is in line with a study using 150 mg/day Pycnogenol^®^ for 6 months in patients with metabolic syndrome (12). GGT is a highly sensitive parameter for liver diseases (44) and it has also been suggested as a predictive biomarker of insufficient cellular antioxidant capacity with low antioxidant defenses being correlated with elevated GGT activity (45). Although a significant reduction in GGT activities was observed in the subgroup of participants who switched from placebo to Pycnogenol^®^ for an additional 3 months, the extent of change was relatively small (23.48 ± 7.90 U/L versus 20.29 ± 4.97 U/L), and clinical relevance is unlikely. A numerically comparable decline occurred in the group continuously taking Pycnogenol^®^ without reaching statistical significance, and no between-group differences were observed at any visit. Throughout the study period, there were no meaningful changes in renal function parameters, both GFR and serum urea remained largely unchanged in both treatment and control groups. This is consistent with earlier clinical trials in which Pycnogenol^®^ showed no appreciable effect on serum urea levels (46, 47).

In our study, leukocyte counts remained stable over the six-month period when patients received Pycnogenol^®^. This is in line with previous work in which no significant changes in leukocyte count were observed among participants who took Pycnogenol^®^ in addition to ramipril for 6 months (48).

In both the Pycnogenol^®^ and placebo group, baseline CRP levels were within the normal range for healthy adults (0.0–0.8 mg/dL) throughout the study. Over the study period, no significant within-group changes were observed in either group. However, between-group comparisons differed significantly at all three visits. This difference was already present at baseline and did not change thereafter, and the baseline-adjusted analysis of the randomized placebo-controlled phase showed no detectable treatment effect (*β* = −0.011; 95% CI = −0.070 to 0.048; *p* = 0.7057). A randomization failure with respect to inflammatory status appears unlikely, since the groups did not differ in the prevalence of inflammatory disease or in analgesic use, both of which were numerically more frequent in the placebo group (Table 1). Given the low absolute concentrations and the absence of any changes over time, the imbalance was most plausibly attributable to the pronounced biological variability (49). A recent meta-analysis demonstrated that Pycnogenol^®^ supplementation significantly reduced CRP levels (50). However, in the studies included in this meta-analysis, baseline CRP levels were notably higher, often exceeding the reference range for healthy adults. In contrast, both study arms in our analysis had baseline CRP levels at the lower end of the reference interval, indicating that previously observed effects of Pycnogenol^®^ on CRP are particularly evident in individuals with elevated baseline levels.

The present study has some limitations. First, not all participants completed the quality-of-life questionnaires throughout the entire study period, resulting in a loss of longitudinal data. However, analyses using all available observations confirmed improvements in the bodily pain and vitality domains of the SF-36v2 in the Pycnogenol^®^ group compared with placebo during the randomized placebo-controlled phase. Second, the reduction in serum phosphate concentrations in the Pycnogenol^®^ group, which reached statistical significance after 6 months during the active-treatment extension, was not investigated mechanistically. In this context, urinary phosphate and calcium excretion were not assessed, which could have provided valuable insight into the underlying mechanisms. Serum iFGF-23 concentrations remained unchanged throughout the study, arguing against an FGF-23-mediated effect. Third, the findings are limited to postmenopausal women with osteopenia or clinically stable osteoporosis who were not receiving pharmacological treatment for osteoporosis. Whether comparable effects occur in men, in patients with severe or progressive disease, or in those receiving anti-osteoporotic medication remains to be established. Fourth, the observation period of 6 months does not allow conclusions on whether the observed improvements in pain and vitality are sustained, or on the long-term safety of continued supplementation. Studies with extended follow-up are therefore warranted. Nevertheless, a major strength of the present study was the comprehensive laboratory evaluation, which included numerous serum parameters beyond those reported in previous Pycnogenol^®^ studies. In addition, the study provided human safety data on the daily administration of 200 mg Pycnogenol^®^ over an observation period of 6 months, which was longer than in earlier investigations of this extract.

## 5 Conclusion

To conclude, this study represents the first randomized, placebo-controlled human trial to examine the safety and impact on quality of life of dietary supplementation with maritime pine bark extract Pycnogenol^®^ in patients with osteopenia or clinically stable osteoporosis. Physiological and unchanged values of laboratory biomarkers including blood count as well as parameters of mineral metabolism, liver and kidney function indicated no major safety signal during a daily intake of 200 mg Pycnogenol^®^ over 6 months in this study population. Furthermore, pain can be highly disruptive to the quality of life (41) in patients with osteoporosis, and participants receiving Pycnogenol^®^ reported improvements in the bodily pain domain of the SF-36v2 during the randomized placebo-controlled phase. Vitality scores were also significantly higher in the Pycnogenol^®^ group than in placebo during this phase. Future research should examine if the observed benefits persist with prolonged use and confirm that Pycnogenol^®^ facilitates maintaining bone health.

## Data Availability

The raw data supporting the conclusions of this article will be made available by the authors, without undue reservation.
