# Supplementary material for: Quality of life and safety of a dietary intervention with French maritime pine bark extract (Pycnogenol®) in women with osteoporotic bone loss: a randomized, placebo-controlled double-blind study—Part II
Source: Front Nutr. 2026 Oct 12;13:1924081. doi: 10.3389/fnut.2026.1924081 (PMC13696226; doi:10.3389/fnut.2026.1924081)
Supplement: Supplementary file 1 [file Table_1.docx]

**Supplementary Table S1**: Outliers identified among clinical laboratory parameters. Data excluded due to outliers employing the ROUT method (Q = 1%).

PYC, Pycnogenol^®^, PLAC, placebo

| Parameter | Baseline | | 3 Months | | 6 Months | |
| --- | --- | --- | --- | --- | --- | --- |
|  | PYC | PLAC | PYC | PLAC | PYC | PYC |
| Calcium | N = 0 | N = 0 | N = 0 | N = 0 | N = 0 | N = 0 |
| Phosphate | N = 0 | N = 0 | N = 0 | N = 0 | N = 0 | N = 0 |
| ALP | N = 2 | N = 0 | N = 2 | N = 0 | N = 0 | N = 0 |
| Vitamin D | N = 0 | N = 0 | N = 0 | N = 0 | N = 1 | N = 0 |
| PTH | N = 0 | N = 0 | N = 1 | N = 0 | N = 0 | N = 0 |
| iFGF-23 | N =1 | N = 0 | N = 2 | N = 0 | N = 4 | N = 0 |
| AST | N = 1 | N = 1 | N = 1 | N = 1 | N = 0 | N = 0 |
| ALT | N = 0 | N = 3 | N = 0 | N = 0 | N = 1 | N = 0 |
| GGT | N = 3 | N = 5 | N = 4 | N = 3 | N = 4 | N = 7 |
| GFR | N = 0 | N = 2 | N = 0 | N = 1 | N = 0 | N = 1 |
| Urea | N = 0 | N = 0 | N = 0 | N = 0 | N = 0 | N = 0 |
| Hemoglobin | N = 0 | N = 0 | N = 0 | N = 0 | N = 0 | N = 0 |
| Leukocytes | N = 2 | N = 0 | N = 2 | N = 0 | N = 2 | N = 0 |
| CRP | N = 3 | N = 8 | N = 4 | N = 9 | N = 4 | N = 4 |

**Supplementary Table S2**: Completeness of the QUALEFFO-41 by domains, treatment group and visit.

PYC, Pycnogenol^®^, PLAC, placebo

| **Domain** | **Questionnaires** | **Baseline** | | **3 Months** | | **6 Months** | |
| --- | --- | --- | --- | --- | --- | --- | --- |
|  |  | PYC | PLAC | PYC | PLAC | PYC | PYC |
| Total Score | Expected | N = 33 | N = 34 | N = 30 | N = 32 | N = 30 | N = 32 |
|  | Completed | N = 29 | N = 32 | N = 25 | N = 27 | N = 21 | N = 29 |
|  | Excluded^1^ Missing | N = 4 | N = 2 | N = 4 | N = 5 | N = 9 | N = 3 |
|  | Excluded^2^ Outlier | N = 0 | N = 0 | N = 1 | N = 0 | N = 0 | N = 0 |
| Pain | Expected | N = 33 | N = 34 | N = 30 | N = 32 | N = 30 | N = 32 |
|  | Completed | N = 29 | N = 32 | N = 26 | N = 27 | N = 21 | N = 29 |
|  | Excluded^1^ Missing | N = 4 | N = 2 | N = 4 | N = 5 | N = 9 | N = 3 |
|  | Excluded^2^ Outlier | N = 0 | N = 0 | N = 0 | N = 0 | N = 0 | N = 0 |
| Activities of daily living | Expected | N = 33 | N = 34 | N = 30 | N = 32 | N = 30 | N = 32 |
|  | Completed | N = 29 | N = 32 | N = 24 | N = 27 | N = 21 | N = 29 |
|  | Excluded^1^ Missing | N = 4 | N = 2 | N = 4 | N = 5 | N = 9 | N = 3 |
|  | Excluded^2^ Outlier | N = 0 | N = 0 | N = 2 | N = 0 | N = 0 | N = 0 |
| Jobs around the house | Expected | N = 33 | N = 34 | N = 30 | N = 32 | N = 30 | N = 32 |
|  | Completed | N = 27 | N = 31 | N = 21 | N = 27 | N = 18 | N = 28 |
|  | Excluded^1^ Missing | N = 4 | N = 2 | N = 4 | N = 5 | N = 9 | N = 3 |
|  | Excluded^2^ Outlier | N = 2 | N = 1 | N = 5 | N = 0 | N = 3 | N = 1 |
| Mobility | Expected | N = 33 | N = 34 | N = 30 | N = 32 | N = 30 | N = 32 |
|  | Completed | N = 26 | N = 31 | N = 25 | N = 27 | N = 21 | N = 29 |
|  | Excluded^1^ Missing | N = 4 | N = 2 | N = 4 | N = 4 | N = 9 | N = 3 |
|  | Excluded^2^ Outlier | N = 3 | N = 1 | N = 1 | N = 1 | N = 0 | N = 0 |
| Leisure and social activities | Expected | N = 33 | N = 34 | N = 30 | N = 32 | N = 30 | N = 32 |
|  | Completed | N = 29 | N = 31 | N = 26 | N = 28 | N = 21 | N = 28 |
|  | Excluded^1^ Missing | N = 4 | N = 2 | N = 4 | N = 4 | N = 9 | N = 3 |
|  | Excluded^2^ Outlier | N = 0 | N = 1 | N = 0 | N = 0 | N = 0 | N = 1 |
| General health perception | Expected | N = 33 | N = 34 | N = 30 | N = 32 | N = 30 | N = 32 |
|  | Completed | N = 29 | N = 32 | N = 26 | N = 28 | N = 21 | N = 28 |
|  | Excluded^1^ Missing | N = 4 | N = 2 | N = 4 | N = 4 | N = 9 | N = 4 |
|  | Excluded^2^ Outlier | N = 0 | N = 0 | N = 0 | N = 0 | N = 0 | N = 0 |
| Mental function | Expected | N = 33 | N = 34 | N = 30 | N = 32 | N = 30 | N = 32 |
|  | Completed | N = 28 | N = 32 | N = 26 | N = 28 | N = 21 | N = 28 |
|  | Excluded^1^ Missing | N = 5 | N = 2 | N = 4 | N = 4 | N = 9 | N = 4 |
|  | Excluded^2^ Outlier | N = 0 | N = 0 | N = 0 | N = 0 | N = 0 | N = 0 |

^1^ Domain score not evaluable because of missing questionnaires or missing item responses, according to the scoring rules described in Methods section

^2^ Data excluded due to outliers (ROUT method, Q = 1%)

**Supplementary Table S3**: Completeness of the SF-36v2 by domains, treatment group and visit.

PYC, Pycnogenol^®^, PLAC, placebo

| **Domain** | **Questionnaires** | **Baseline** | | **3 Months** | | **6 Months** | |
| --- | --- | --- | --- | --- | --- | --- | --- |
|  |  | PYC | PLAC | PYC | PLAC | PYC | PYC |
| Physical functioning | Expected | N = 33 | N = 34 | N = 30 | N = 32 | N = 30 | N = 32 |
|  | Completed | N = 28 | N = 32 | N = 28 | N = 25 | N = 22 | N = 29 |
|  | Excluded^1^ Missing | N = 5 | N = 2 | N = 2 | N = 4 | N = 8 | N = 3 |
|  | Excluded^2^ Outlier | N = 0 | N = 0 | N = 0 | N = 3 | N = 0 | N = 0 |
| Physical role functioning | Expected | N = 33 | N = 34 | N = 30 | N = 32 | N = 30 | N = 32 |
|  | Completed | N = 25 | N = 30 | N = 27 | N = 26 | N = 21 | N = 29 |
|  | Excluded^1^ Missing | N = 8 | N = 4 | N = 3 | N = 6 | N = 9 | N = 3 |
|  | Excluded^2^ Outlier | N = 0 | N = 0 | N = 0 | N = 0 | N = 0 | N = 0 |
| Bodily Pain | Expected | N = 33 | N = 34 | N = 30 | N = 32 | N = 30 | N = 32 |
|  | Completed | N = 28 | N = 31 | N = 28 | N = 28 | N = 22 | N = 28 |
|  | Excluded^1^ Missing | N = 5 | N = 3 | N = 2 | N = 4 | N = 8 | N = 4 |
|  | Excluded^2^ Outlier | N = 0 | N = 0 | N = 0 | N = 0 | N = 0 | N = 0 |
| General health perception | Expected | N = 33 | N = 34 | N = 30 | N = 32 | N = 30 | N = 32 |
|  | Completed | N = 27 | N = 31 | N = 28 | N = 28 | N = 21 | N = 27 |
|  | Excluded^1^ Missing | N = 6 | N = 3 | N = 2 | N = 4 | N = 9 | N = 5 |
|  | Excluded^2^ Outlier | N = 0 | N = 0 | N = 0 | N = 0 | N = 0 | N = 0 |
| Vitality | Expected | N = 33 | N = 34 | N = 30 | N = 32 | N = 30 | N = 32 |
|  | Completed | N = 28 | N = 31 | N = 26 | N = 28 | N = 22 | N = 28 |
|  | Excluded^1^ Missing | N = 5 | N = 3 | N = 3 | N = 4 | N = 8 | N = 4 |
|  | Excluded^2^ Outlier | N = 0 | N = 0 | N = 1 | N = 0 | N = 0 | N = 0 |
| Social role functioning | Expected | N = 33 | N = 34 | N = 30 | N = 32 | N = 30 | N = 32 |
|  | Completed | N = 28 | N = 31 | N = 27 | N = 28 | N = 22 | N = 28 |
|  | Excluded^1^ Missing | N = 5 | N = 3 | N = 2 | N = 4 | N = 8 | N = 4 |
|  | Excluded^2^ Outlier | N = 0 | N = 0 | N = 1 | N = 0 | N = 0 | N = 0 |
| Emotional role functioning | Expected | N = 33 | N = 34 | N = 30 | N = 32 | N = 30 | N = 32 |
|  | Completed | N = 21 | N = 30 | N = 22 | N = 28 | N = 16 | N = 29 |
|  | Excluded^1^ Missing | N = 7 | N = 4 | N = 3 | N = 4 | N = 9 | N = 3 |
|  | Excluded^2^ Outlier | N = 5 | N = 0 | N = 5 | N = 0 | N = 5 | N = 0 |
| Mental health | Expected | N = 33 | N = 34 | N = 30 | N = 32 | N = 30 | N = 32 |
|  | Completed | N = 27 | N = 31 | N = 27 | N = 26 | N = 22 | N = 28 |
|  | Excluded^1^ Missing | N = 6 | N = 3 | N = 3 | N = 5 | N = 8 | N = 4 |
|  | Excluded^2^ Outlier | N = 0 | N = 0 | N = 0 | N = 1 | N = 0 | N = 0 |

^1^ Domain score not evaluable because of missing questionnaires or missing item responses, according to the scoring rules described in Methods section

^2^ Data excluded due to outliers (ROUT method, Q = 1%)

**Supplementary Table S4:** QUALEFFO-41 scores recorded by the study participants. In the QUALEFFO-41 questionnaire, lower scores signify a higher quality of life_._

| **Domain** | **Measurement**  **Time** | **Pycnogenol^®^**  **Mean ± SD n ^1^** | **Placebo^2^**  **Mean ± SD n ^1^** | ***p*-value ^b^** | **Mixed-effects model**  ***p*-values ^c^** |
| --- | --- | --- | --- | --- | --- |
| Activities of daily living | Baseline (V1) | 12.95 ± 9.73 29 | 17.41 ± 11.20 32 | 0.1013 | time: 0.1540  group: 0.0982  time × group: 0.9725 |
|  | 3 months (V3)  *p*-value ^a^ vs. V1 | 9.15 ± 7.37 24  0.4025 | 14.14 ± 11.44 27  0.1333 | 0.0674 |  |
|  | 6 months (V5)  *p*-value ^a^ vs. V1  *p*-value ^a^ vs. V3 | 12.89 ± 11.44 21  0.7802  0.8644 | 16.40 ± 12.87 29  0.7308  0.8367 | 0.3148 |  |
| Jobs around the house | Baseline (V1) | 9.44 ± 12.27 27 | 17.26 ± 20.03 31 | 0.0754 | time: 0.9242  group: 0.0251 *  time × group: 0.4467 |
|  | 3 months (V3)  *p*-value ^a^ vs. V1 | 4.76 ± 6.80 21  0.4200 | 19.63 ± 24.96 27  0.3619 | 0.0059 * |  |
|  | 6 months (V5)  *p*-value ^a^ vs. V1  *p*-value ^a^ vs. V3 | 7.50 ± 10.04 18  0.9898  0.7297 | 16.89 ± 19.44 28  0.9577  0.9352 | 0.0375 * |  |
| Baseline-adjusted analysis, V1 → V3 ^d^; β (group) = -4.21; 95% CI = -8.76 to 0.35; *p* = 0.0692; n = 47 | | | |  |  |
| Mobility | Baseline (V1) | 10.27 ± 10.76 26 | 14.55 ± 16.31 31 | 0.2405 | time: 0.7242  group: 0.6846  time × group: 0.4462 |
|  | 3 months (V3)  *p*-value ^a^ vs. V1 | 11.26 ± 11.09 25  0.9852 | 12.46 ± 14.10 27  0.6294 | 0.7329 |  |
|  | 6 months (V5)  *p*-value ^a^ vs. V1  *p*-value ^a^ vs. V3 | 13.55 ± 14.43 21  0.5588  0.8759 | 14.22 ± 15.01 29  0.7667  0.9251 | 0.8738 |  |
| Leisure and social activities | Baseline (V1) | 20.49 ± 18.38 29 | 21.25 ± 20.90 31 | 0.8827 | time: 0.4124  group: 0.4287  time × group: 0.5158 |
|  | 3 months (V3)  *p*-value ^a^ vs. V1 | 16.79 ± 16.57 26  0.4003 | 18.53 ± 21.35 28  0.9393 | 0.7378 |  |
|  | 6 months (V5)  *p*-value ^a^ vs. V1  *p*-value ^a^ vs. V3 | 17.64 ± 18.03 21  0.2146  0.9520 | 20.95 ± 19.81 28  0.9151  0.2533 | 0.5456 |  |
| General health perception | Baseline (V1) | 47.55 ± 22.42 29 | 55.86 ± 19.59 32 | 0.1305 | time: 0.5586  group: 0.1404  time × group: 0.7450 |
|  | 3 months (V3)  *p*-value ^a^ vs. V1 | 47.16 ± 19.62 26  0.9991 | 52.08 ± 20.86 28  0.5935 | 0.3762 |  |
|  | 6 months (V5)  *p*-value ^a^ vs. V1  *p*-value ^a^ vs. V3 | 46.42 ± 20.85 21  0.6863  0.9944 | 53.58 ± 22.26 28  0.7603  0.8861 | 0.2541 |  |
| Mental function | Baseline (V1) | 30.18 ± 16.32 28 | 38.78 ± 16.52 32 | 0.0475 * | time: 0.2733  group: 0.0550  time × group: 0.6199 |
|  | 3 months (V3)  *p*-value ^a^ vs. V1 | 28.85 ± 17.29 26  0.6803 | 34.37 ± 11.91 28  0.6574 | 0.1818 |  |
|  | 6 months (V5)  *p*-value ^a^ vs. V1  *p*-value ^a^ vs. V3 | 32.61 ± 15.27 21  0.8289  0.0940 | 37.10 ± 19.21 28  0.7166  0.7974 | 0.3668 |  |
|  | ^1^ Number of participants with an evaluable domain score at the respective visit, after exclusion of incomplete questionnaires according to the scoring rules described in the Methods section and of outliers by the ROUT procedure (Q = 1%); n therefore varies between visits. ^2^ 6-month value: Switch to Pycnogenol^®^.  Values are observed means ± SD. All *p-*values derive from one mixed-effects model (REML) per domain, with treatment group, time and their interaction as fixed effects and participant as a random effect, using all available observations. Model estimates are therefore not derivable from the descriptive values shown.  **^a^** Within-group comparisons versus V1 or V3; **^b^** between-group comparison at that visit, unadjusted for baseline. All pairwise comparisons were corrected for multiplicity using Tukey’s method. **^c^** Overall *p-*values for the fixed effects of the model. From V3 onward all participants received Pycnogenol^®^, so between-group comparisons at V5 reflect duration of exposure rather than treatment allocation and are descriptive. **^d^** Multiple linear regression for the randomized phase (V3 score as dependent variable, V1 score and treatment group as independent variables), corresponding to an analysis of covariance (ANCOVA). Performed for domains showing a between-group difference at V3. β (group) is Pycnogenol^®^ minus placebo, with 95% CI by profile likelihood; participants with evaluable scores at both V1 and V3.  * Significant *p*-value for α < 0.05. | | | | |

**Supplementary Table S5:** SF-36 v2 scores recorded by the study participants. In the SF-36v2 questionnaire, higher scores indicate a higher quality of life_._

| **Domain** | **Measurement**  **Time** | **Pycnogenol^®^**  **Mean ± SD n ^1^** | **Placebo^2^**  **Mean ± SD n ^1^** | ***p*-value ^b^** | **Mixed-effects model**  ***p*-values ^c^** |
| --- | --- | --- | --- | --- | --- |
| Physical functioning | Baseline (V1) | 72.71 ± 24.37 28 | 73.76 ± 26.63 32 | 0.8745 | time: 0.2000  group: 0.8625  time × group: 0.8398 |
|  | 3 months (V3)  *p*-value ^a^ vs. V1 | 76.11 ± 22.76 28  0.7115 | 79.40 ± 23.69 25  0.9764 | 0.6091 |  |
|  | 6 months (V5)  *p*-value ^a^ vs. V1  *p*-value ^a^ vs. V3 | 73.18 ± 27.80 22  0.6564  0.4424 | 70.34 ± 27.71 29  0.6387  0.8888 | 0.7194 |  |
| Physical role functioning | Baseline (V1) | 73.00 ± 36.74 25 | 55.83 ± 45.34 30 | 0.1269 | time: 0.2246  group: 1268  time × group: 0.5989 |
|  | 3 months (V3)  *p*-value ^a^ vs. V1 | 68.52 ± 43.07 27  0.8576 | 68.27 ± 41.57 26  0.8079 | 0.9830 |  |
|  | 6 months (V5)  *p*-value ^a^ vs. V1  *p*-value ^a^ vs. V3 | 65.48 ± 43.64 21  0.8506  0.5863 | 50.00 ± 44.32 29  0.7694  0.1747 | 0.2255 |  |
| General health perception | Baseline (V1) | 62.48 ± 23.62 27 | 52.19 ± 17.78 31 | 0.0702 | time: 0.3680  group: 0.0919  time × group: 0.3999 |
|  | 3 months (V3)  *p*-value ^a^ vs. V1 | 64.71 ± 23.66 28  0.9971 | 57.14 ± 19.27 28  0.8634 | 0.1949 |  |
|  | 6 months (V5)  *p*-value ^a^ vs. V1  *p*-value ^a^ vs. V3 | 57.62 ± 21.56 21  0.6222  0.4792 | 54.85 ± 18.91 27  0.9073  0.9745 | 0.6444 |  |
| Social role functioning | Baseline (V1) | 79.46 ± 24.11 28 | 72.18 ± 26.36 31 | 0.2721 | time: 0.6241  group: 0.1689  time × group: 0.8701 |
|  | 3 months (V3)  *p*-value ^a^ vs. V1 | 83.33 ± 18.67 27  0.9944 | 75.89 ± 24.28 28  0.9750 | 0.2075 |  |
|  | 6 months (V5)  *p*-value ^a^ vs. V1  *p*-value ^a^ vs. V3 | 79.55 ± 24.86 22  0.7562  0.5006 | 71.43 ± 23.29 28  0.9786  0.8947 | 0.2451 |  |
| Emotional role functioning ^e^ | Baseline (V1) | 100.00 ± 0.00 21 | 62.22 ± 42.65 30 | < 0.0001 * | time: 0.3491  group: < 0.0001 *  time × group: 0.3491 |
|  | 3 months (V3)  *p*-value ^a^ vs. V1 | 100.00 ± 0.00 22  - | 78.58 ± 34.20 28  0.5139 | 0.0026 * |  |
|  | 6 months (V5)  *p*-value ^a^ vs. V1  *p*-value ^a^ vs. V3 | 100.00 ± 0.00 16  -  - | 68.97 ± 39.78 29  0.7911  0.5647 | 0.0002 * |  |
| Baseline-adjusted analysis, V1 → V3 ^d^; β (group) = 15.13; 95% CI = -3.46 to 33.72; *p* = 0.1078; n = 43 | | | |  |  |
| Mental health | Baseline (V1) | 75.11 ± 14.65 27 | 67.35 ± 18.19 31 | 0.0777 | time: 0.6442  group: 0.0664  time × group: 0.3108 |
|  | 3 months (V3)  *p*-value ^a^ vs. V1 | 77.19 ± 15.48 27  0.9340 | 71.23 ± 11.54 26  0.9969 | 0.1181 |  |
|  | 6 months (V5)  *p*-value ^a^ vs. V1  *p*-value ^a^ vs. V3 | 71.82 ± 16.99 22  0.4302  0.4352 | 69.29 ± 17.99 28  0.9098  0.6864 | 0.6126 |  |
|  | ^1^ Number of participants with an evaluable domain score at the respective visit, after exclusion of incomplete questionnaires according to the scoring rules described in the Methods section and of outliers by the ROUT procedure (Q = 1%); n therefore varies between visits. ^2^ 6-month value: Switch to Pycnogenol^®^.  Values are observed means ± SD. All *p-*values derive from one mixed-effects model (REML) per domain, with treatment group, time and their interaction as fixed effects and participant as a random effect, using all available observations. Model estimates are therefore not derivable from the descriptive values shown.  **^a^** Within-group comparisons versus V1 or V3; **^b^** between-group comparison at that visit, unadjusted for baseline. All pairwise comparisons were corrected for multiplicity using Tukey’s method. **^c^** Overall *p-*values for the fixed effects of the model. From V3 onward all participants received Pycnogenol^®^, so between-group comparisons at V5 reflect duration of exposure rather than treatment allocation and are descriptive. **^d^** Multiple linear regression for the randomized phase (V3 score as dependent variable, V1 score and treatment group as independent variables), corresponding to an analysis of covariance (ANCOVA). Performed for domains showing a between-group difference at V3. β (group) is Pycnogenol^®^ minus placebo, with 95% CI by profile likelihood; participants with evaluable scores at both V1 and V3. ^e^ After outlier removal, all remaining values in the Pycnogenol^®^ group were at the maximum score, leaving no variance. Comparisons in this domain are not interpretable and are shown for completeness only.  * Significant *p*-value for α < 0.05. | | | | |
